# Recent Advances in Single-Cell View of Mesenchymal Stem Cell in Osteogenesis

**DOI:** 10.3389/fcell.2021.809918

**Published:** 2022-01-05

**Authors:** Fangyuan Shen, Yu Shi

**Affiliations:** State Key Laboratory of Oral Diseases and National Clinical Research Center for Oral Diseases, West China Hospital of Stomatology, Sichuan University, Chengdu, China

**Keywords:** mesenchymal stem cells, osteogenesis, lineage tracing, single-cell, niche

## Abstract

Osteoblasts continuously replenished by osteoblast progenitor cells form the basis of bone development, maintenance, and regeneration. Mesenchymal stem cells (MSCs) from various tissues can differentiate into the progenitor cell of osteogenic lineage and serve as the main source of osteoblasts. They also respond flexibly to regenerative and anabolic signals emitted by the surrounding microenvironment, thereby maintaining bone homeostasis and participating in bone remodeling. However, MSCs exhibit heterogeneity at multiple levels including different tissue sources and subpopulations which exhibit diversified gene expression and differentiation capacity, and surface markers used to predict cell differentiation potential remain to be further elucidated. The rapid advancement of lineage tracing methods and single-cell technology has made substantial progress in the characterization of osteogenic stem/progenitor cell populations in MSCs. Here, we reviewed the research progress of scRNA-seq technology in the identification of osteogenic markers and differentiation pathways, MSC-related new insights drawn from single-cell technology combined with experimental technology, and recent findings regarding the interaction between stem cell fate and niche in homeostasis and pathological process.

## 1 Introduction

The bone formation depends on the activation and recruitment of osteogenic stem/progenitor cells during bone development, reconstruction, and fracture repair. During embryogenesis, mesoderm-derived limb bud mesenchymal progenitors (LMPs) differentiate into osteochondrogenic lineages and generate primitive cartilage templates. After LMPs differentiate into cartilage, long bones are built through endochondral ossification. At the beginning of primary ossification center formation, perichondrial progenitor cells and blood vessels extend into cartilage lacuna left by the degeneration of chondrocytes in the cartilage template, and then new bone marrow is formed (Maes et al., 2010; Ono et al., 2014). In postnatal life, bone is a dynamic tissue that is constantly being resorbed and remodeled. The undifferentiated MSCs in the bone marrow stroma are ancestors of osteoprogenitor cells and pre-osteoblasts, and under the regulation of Runt-related transcription factor 2 (*Runx2*) and Osterix (*Osx*) (Nakashima et al., 2002; Lian et al., 2006), they differentiate into mature osteoblasts which have a limited lifespan and are constantly replenished by osteogenic precursor cells.

MSCs have long been regarded as a direct source of osteogenic lineage progenitor cells in bone tissue. Their source is complex and correlates with stage and tissue specificity. MSC can be isolated from tissues such as teeth, bone marrow, adipose, umbilical cord, etc. The distribution, differentiation direction, immunosuppressive ability of MSCs subgroups from different sources are various, and the expression pattern also changes with age. Extensive studies have shown that MSCs are heterogeneous mixtures of multiple stem cell lineages, which can differentiate into osteoblasts, chondrocytes, and bone marrow stromal cells, fat cells, muscle cells, and endothelial cells (Uccelli et al., 2008; Chen et al., 2019). To identify cell subsets with specific functions in heterogeneous MSCs, cell surface markers are continuously explored. However, the non-negligible heterogeneities and lack of stage-specific markers hindered the identification and positioning of cell types and formed a significant barrier to our understanding of MSC populations. Until 2018, single-cell and lineage tracing technology was used to identify self-renewing and multipotent skeletal stem cells (SSCs) which could only differentiate into the progenitor of the osteogenic lineage (osteoprogenitors, chondroprogenitors, and progenitor cells of stroma) (Chan et al., 2018). With the emergence of numerous MSC-related studies, researchers have meticulously named the subgroups of MSCs such as embryonic Skeletal Stem/progenitor Cell (eSSPC), Bone Marrow Mesenchymal Cell (BMSC), Periosteal Stem Cell (PSC), etc., based on tissue origin, the developmental stage of donors, and differentiation characteristics. Possessing functional properties consistent with MSC, these renamed subgroups belong to the category of MSC.

Due to the rapid progress of mouse lineage-tracking techniques and single-cell RNA sequencing (scRNA-seq), researchers have made extraordinary progress in identifying and characterizing MSC heterogeneity and reconstructing osteogenic regulatory networks. To date, MSCs have been detected in growth plate cartilage, bone marrow stroma, the superficial layer of meniscus, bone surface resting-state bone lining cells and specialized fibroblasts in the craniofacial structure and have been characterized by several markers (i.e., leptin receptor [*LepR*], cathepsin K [*Ctsk*], glioma-associated oncogene homolog 1 [*Gli1*], and platelet-derived growth factor receptor [*Pdgfr*], etc.) *in vivo* (Maruyama et al., 2016; Yue et al., 2016; Shi et al., 2017; Mizuhashi et al., 2018; Baryawno et al., 2019; Bohm et al., 2019; Ortinau et al., 2019; Ponte et al., 2020; Wei et al., 2021; Zhang et al., 2021). In this review, we summarized the heterogeneity of MSCs derived from limb buds and postnatal distinct hard tissues as determined by single-cell resolution studies and the biologic functions and characteristics of new labeled stem/progenitor subpopulations. We also focused on the research progress on the osteogenic subpopulation of cre-targeted MSCs and the interaction between the osteogenic niche and precursor cells.

## 2 Traditional MSCs Identification Methods

**FIGURE 1:**
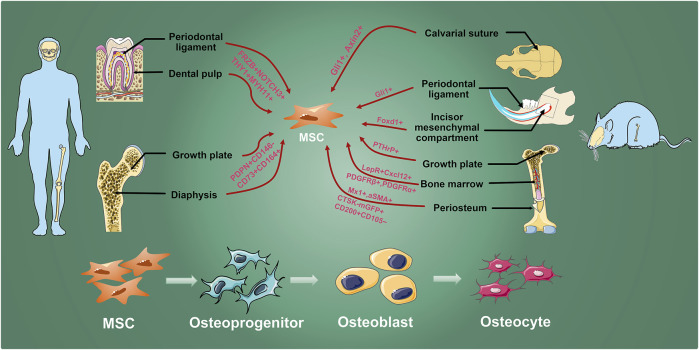
MSC inhabit in various hard tissues. Osteogenic MSCs which are labeled by researchers with different genes exist in human molars and long bones, and mouse calvarial sutures, incisors, molars, and long bones. They are finally differentiated into osteocyte through osteoprogenitors and osteoblasts to involve in maintaining bone homeostasis, growth, development, and injury repair.

Current knowledge of MSCs mainly originates from experiments with human and rodent bone marrow cells. *In vitro* cultivation and allogeneic cell transplantation have become the gold standard for identifying MSC. Early studies verified the adherence, the ability to form fibroblast colonies (CFU-F), and the osteogenic ability after allograft transplantation to identify the proliferation and trilineage differentiation potential of MSCs (Cao et al., 2020). Flow cytometry was used to screen cell surface markers (L. Ramos et al., 2016). In recent years, many studies have begun to use lineage-tracing techniques to track the activity of MSC maker+ cells in transgenic mice, thereby identifying a series of MSC markers, such as *Grem1* (Worthley et al., 2015), *Gli1* (Shi et al., 2017), *LepR* (Zhou et al., 2014), *Pthrp* (Mizuhashi et al., 2018), *Sox9* (Kuwahara et al., 2019), *Cxcl12* (Matsushita et al., 2020), *Prx1* (Moore et al., 2018), etc. However, the proposed surface markers generally have the problem of low specificity, and as the physiological state and the microenvironment change, MSC markers will alter accordingly. With source diversity, MSCs can be isolated from hard tissues such as teeth, bone marrow, and craniofacial sutures. There is evidence that MSCs from different tissues are divergent (Figure 1) (Sharpe, 2016; Huang et al., 2019; Zhou et al., 2019). Although different tissue-derived MSC all meet the minimum standards for defining MSCs, their transcriptome pattern and multipotential differentiation capacity may also be vastly different (Table 1). Therefore, based on the complex expression alteration of MSC, a single surface marker is not enough to describe the heterogeneous MSC population.

**TABLE 1:** Characterization of embryonic and hard tissue-derived stem/progenitor cell (WPC, weeks post conception; yo, years old).

Species	Localization	Age	Cell	Marker	Reference
Human	Limb buds	5 WPC	Osteo-chondrogenic progenitorss	SOX9^low^PDGFRα^hi^	He et al. (2021a)
	Limb bud long bones	8 WPC	Embryonic skeletal stem/progenitor cells	PDGFRα^low/–^PDPN^+^CADM1^+^	He et al. (2021a)
	Embryonic calvarium	8 WPC	Neural crest-derived cells	PDGFRα^low/–^PDPN^+^CADM1^+^	He et al. (2021a)
	Third molar dental pulp and periodontal ligament	18–35yo	Mesenchymal stem cells	FRZB^+^NOTCH3^+^THY1^+^ MYH11^+^	Pagella et al. (2021)
	Femur growth plate and diaphysis	17 weeks fetal	Human-skeletal stem cells	PDPN^+^CD146^–^CD73^+^CD164^+^	Chan et al. (2018)
Mice	Forelimb buds	E10.5-E10.75	Osteo-chondrogenic progenitors	Sox9^+^PDGFRα^hi^	Reinhardt et al. (2019)
	Forelimb buds	E10.5-E10.75	Transition betweenlimb bud mesenchymal progenitors and osteo-chondrogenic progenitors	Sox9^–^PDGFRα^hi^	Reinhardt et al. (2019)
	Forelimb buds	E10.5-E10.75	Limb bud mesenchymal progenitors	Sox9^–^JAG1^+^	Reinhardt et al. (2019)
	Hind limb	E12.5	Musculoskeletal stem cells	Scx^+^Hoxd13^+^	Yin et al. (2020)
	Femur	Postnatal	Mouse skeletal stem cells	CD45^–^Ter119^–^Tie^–^AlphaV^+^Thy^–^6C3^–^CD105^–^CD200^+^	Chan et al. (2015)
	Incisor mesenchymal compartment near the labial cervical loop	2–4 months	Mesenchymal stem cells	Foxd1^+^	Krivanek et al. (2020)
	Molar periodontal ligament apical	Adult	Periodontal ligament stem cells	Gli1^+^	Men et al. (2020)
	The resting zone of growth plate	Postnatal	Skeletal stem cells	PTHrP^+^	Mizuhashi et al. (2018)
	The periphery of the growth plate immediately adjacent to the perichondrium	Fetal and neonatal	Mesenchymal precursor cells/chondrocytes	PTHrP^+^	Mizuhashi et al. (2019)
	Metaphysis and diaphysis	3 week	Mesenchymal stromal cells from the metaphysis and diaphysis	PDGFRα^+^, PDGFRβ^+^	Sivaraj et al. (2021)
	Metaphysis and diaphysis	Postnatal	Bone marrow stromal cells	LepR^+^	Shu et al. (2021)
	Periosteum in the metaphysis and diaphysis	Adult	Periosteal skeletal stem cells	Mx1^+^, aSMA^+^	Sivaraj et al. (2021)
	Bone marrow stromal of femur and tibia	6–8 weeks	Mesenchymal stem cells	LepR^+^ Cxcl12^+^	Baryawno et al. (2019)
	Long bone and calvarium	Postnatal	Periosteal stem cells	CTSK-mGFP^+^CD200^+^CD105^−^	Debnath et al. (2018)

## 3 scRNA-Seq-Based MSC Identification and Function Assessment

**FIGURE 2:**
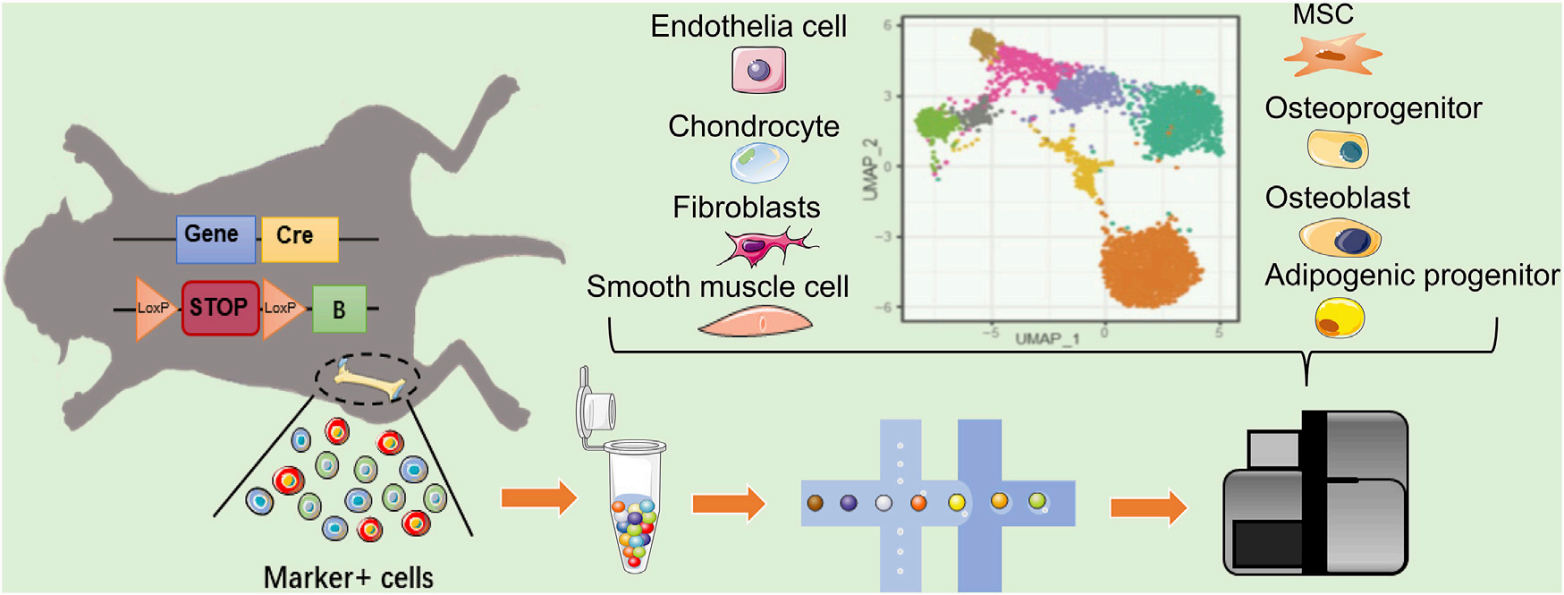
Single cell reveals the heterogeneity of MSCs markers targeting cells. In mice with Cre–loxP recombination system, the MSC markers gene expressing-cells will be fluorescently labeled. When two loxP sites exist in the same DNA strand with the same orientation, terminator between two loxP will be removed by Cre, allowing the fluorescent protein to be expressed. The heterogeneity of labeled MSCs can revealed by scRNA-seq. In addition to MSC, the existing MSC marker-labeled cell population may include Osteo- and adipo-lineage cells, chondrocytes, epithelial cells, and fibroblast smooth muscle cells, etc. Blue square: MSC marker gene; yellow square: cyclization recombination enzyme gene; red rounded rectangle: stop codon; orange triangle: specific recognition site of Cre recombinase; green square: fluorescent reporter.

scRNA-seq profile gene expression at single-cell resolution, which is ideally suited to explore the heterogeneity of MSCs. The key to scRNA-seq technology is single-cell isolation and independent library construction. Using droplet-based microfluidics technology, single cell and gel beads containing a barcode, unique molecular index, primers, and enzymes were wrapped in an oil droplet through a microfluidic chip (Zhou et al., 2021). The barcode in each oil droplet is a unique DNA sequence, thus allowing the distinction of the source of the target sequence during sequencing (Figure 2). Therefore, researchers achieve large-scale single-cell isolation and routine profiling of thousands of cells via constructing libraries at one time. The scRNA-seq data set analysis of the MSC population roughly follows three main steps (Andrews et al., 2021). First, after quality control and normalization, the cells are divided into multiple subpopulations through dimensionality reduction and unsupervised clustering. Subpopulations are assigned cell types based on their gene expression patterns and prior knowledge. Second, analysis of cell heterogeneity within each cell type can identify MSC subpopulations with distinct cell states and expression programs. Genes that are differentially expressed between subpopulations can be regarded as potential markers. Third, among MSC-related studies, single-cell sequencing objects are typically a heterogeneous cell population, which emerges from the development or differentiation process of pluripotent stem cells making fate decisions and transitioning to specific cell types through intermediate cell states. Pseudotime inference constructs a pseudo-temporal process trajectory to order cells based on the gradual transition of transcriptomes. In MSC-related cases, these trajectories measure the relative progression of each cell along the development or differentiation process, allowing us to understand the pseudo-temporal behavior without explicit time series data (Campbell and Yau, 2018; Herring et al., 2018). Using scRNA-seq, researchers could subdivide MSCs population according to transcription information of individual cells, and predict potential gene markers to label cell populations by differential analysis, which is quite beneficial for finding suitable MSCs groups with different clinical needs of the stem cell therapy.

The recently emerging single-cell epigenome-, genome-, and proteome-sequencing technologies also provide promising directions (Nam et al., 2021). Li et al. (2018) team systematically describes the dynamic changes of the development of mouse preimplantation embryos from five epigenome levels (chromatin status, DNA methylation, copy number variation, and ploidy). Ai et al. (2019) and Wang et al. (2019) used independently developed CoBATCH and sc-itChIP technologies to profile the heterogeneity of endothelial lineage development, differentiation, and function in ten different tissues of mouse embryos. Chaligne et al. (2021) combined single-cell sequencing of DNA methylation, scRNA-seq and targeted genotyping to analyze diffuse gliomas, revealing dysregulated epigenetic mechanisms underlying gliomagenesis. Single-cell multi-omics integrated analysis is a powerful tool to study stem cell development, but there is still a gap in MSC-related research, and remains an uncovered space for future research.

### 3.1 Identification of MSCs in Embryonic and Fetal Limb Buds

In the initial stage of limb bud development, LMPs are composed of distinct progenitor cell types, which show heterogeneous characteristics and differentiated states (Table 1) (Norrie et al., 2014; Ornitz and Marie, 2015; Tickle and Towers, 2017). *SOX9*-expressing LMPs differentiate into cartilage and are regarded as osteochondrogenic progenitors (OCPs) (Akiyama et al., 2005), which generate primitive cartilage templates and then form limb bones through endochondral ossification. In contrast, another method of bone formation, intramembranous ossification builds the most of craniomaxillofacial bones. During this process, bone develops directly from sheets of mesenchymal tissue without the formation of intermediate cartilage.

In 2019, Reinhardt et al. (2019) identified the molecular characterization of various mesenchymal progenitor subpopulations in the early forelimb buds of mice (E10.5‒E10.75). They found that *SOX9* ^−^ *JAG1* ^+^ cells that resided in the distal posterior of mesenchyme were the most immature progenitor cells, which mainly depended on Sonic Hedgehog and apical ectodermal ridge-Fibroblast Growth Factor signaling to maintain their proliferative and undifferentiated state, through *Grem1*-mediated Bone Morphogenetic Protein (BMP) antagonism to avoid BMP-induced apoptosis (Michos et al., 2004). In contrast, *SOX9* ^+^ *PDGFRα* ^hi^ OCPs are sensitive to BMP signaling in the mesenchyme core where limb buds will initially form, while changing from proliferative signals to responding to differentiation signaling. This kind of signal response transformation is a necessary condition for transformation from LMPs to OCPs. As a transition between *SOX9* ^−^ *JAG1* ^+^ cells and *SOX9* ^+^ *PDGFRα* ^hi^ OCPs, *SOX*9^−^ *PDGFRα* ^hi^ cells highly express T-Box transcription factor 2 (*TBX2*) which is involved in the repression of *Grem1*, enhancing the activity of BMP signals and endow LMPs with the potential to form limb buds (Michos et al., 2004; Farin et al., 2013). For murine hindlimb, Yin et al. (2020) named a cluster, composed predominantly of E12.5 cells which highly express scleraxis (*Scx*) and homeobox protein hox-D13 (*Hoxd13*) as musculoskeletal stem cells. *Scx* ^+^ musculoskeletal stem cells can generate soft tissue (myocytes, meniscus cells, and tenocytes) and hard tissue (chondrocytes and osteocytes) progenitors, marked by *Scx* ^*+*^ *Col1a1* ^*+*^ and *Scx* ^*+*^ *Sox9* ^*+*^ respectively. However, Kelly et al. (2020) observed faint and scattered expression of *Scx* at a time point after E13.5 and no expression in E11.5 and E13.5, and did not detect clusters marked by *Scx* from scRNA-seq analysis. The peak of *Scx* expression appeared in the middle of the trajectory (around E12.5). It has been reported, although *Scx* is transiently expressed in the chondrogenic lineage and entheseal cartilage, it is particularly important for the correct integration of musculoskeletal components (Blitz et al., 2013; Zelzer et al., 2014; Killian and Thomopoulos, 2016). Therefore, we speculated that *Scx*, a key transcription factor that regulates musculoskeletal tissue morphogenesis, may be only transiently expressed around E12.5, but the mechanism of *Scx* action remains unknown, and needs to be further investigated.

Human embryonic cells continuously differentiate from limb bud mesenchymal progenitor cells into OCPs, which gives rise to osteogenic and chondrogenic lineages. He et al. (2021a) identified four mesenchymal cell subpopulations (Limb Bud Mesenchyme1-3 and OCP) in human limb buds at 5 weeks post-conception (WPC). Similar to mouse OCPs, human limb bud OCPs (*SOX9* ^low^ *PDGFRα* ^hi^) were located in the core mesenchyme and possessed chondrogenic potential. At 8WPC, a cluster marked by cell adhesion molecule 1 (*CADM1*) was assigned as perichondral embryonic skeletal stem/progenitor cells (eSSPCs); these cells are self-renewable and capable of generating osteochondral lineage. At the same developmental stage, a group of neural crest-derived stem/progenitor cells shared immunophenotype and transcriptional network as long bone-derived eSSPC were identified in the calvaria sagittal suture. They were characterized by the gene expression signature of intramembranous ossification, which mediates the development of the human craniofacial skeleton. In 2018, Chan et al. (2018) revealed markers of human SSC (*PDPN*+*CD146*-*CD73* ^+^ *CD164*+) via scRNA-seq performed on a 17-week fetal femur growth plate and diaphysis. The isolated human SSCs displayed self-renewal abilities *in vitro* and could generate multilineage ossicles composed of bone, cartilage, and stromal progenitors under the renal capsule of NSG mice *in vivo*.

### 3.2 Hard Tissue-Derived MSCs in Postnatal Stages

Limb bones and MSCs residing in them primarily originate from the lateral plate mesoderm during embryogenesis, which create bone tissue by means of endochondral ossification. Neural crest cells located in the neuroectoderm are the principal source of craniofacial bones, cartilage and teeth. The neural crest-derived MSCs residing in the craniofacial region possess characteristics of multidirectional differentiation and low immunogenicity, which are comparable with the mesoderm-derived MSC, but the neural crest-derived MSCs are mostly involved in endochondral ossification.

#### 3.2.1 Dental-Derived MSCs

Growing mouse incisors contains a continuously replenished mesenchymal compartment composed of dentin-secreting odontoblasts and various types of pulp cells. Krivanek et al. (2020) used single-cell technology to study the heterogeneity of the mesenchymal compartment in mouse incisors. They found a apical pulp subtype marked by *Smoc2* and *Sfrp2*, which was specifically located in the apical pulp area in cervical loops and included potential stem/progenitor cells that express genes related to self-renewal such as *Gli1* and *Thy1*. *Foxd1* ^+^ multipotent MSCs which mainly generated periodontoblastic pulp cells and odontoblasts were screened out, and *Foxd1*-traced cells were only detected in the mesenchymal compartment adhering to the labial cervical loop, revealing the pluripotency of *Foxd1* ^+^ MSCs and spatially constrained structure of the self-renew in growing mouse incisors. By comparing the cell subtypes of human apical and mouse distal incisor pulp, they inferred that although cell types are similar, evolutionary differences in gene expression programs that regulate development and homeostasis of dental pulp excluded the possibility of establishing precisely homologous subpopulation in the dental pulp between mouse and human (Krivanek et al., 2020). In adult mouse molar periodontal ligament, *Gli1* ^+^ cells enriched in the apical region are regarded as periodontal ligament stem cells, which can generate periodontal ligament, alveolar bone, and cementum, under Wnt signaling-mediated regulation (Men et al., 2020). Pagella et al. (2021) marked MSCs by highly expressed *FRZB*, *NOTCH3*,*THY1*, and *MYH11* in the dental pulp and periodontium of human third molars. In addition, *CCL2* and collagen-encoding genes were significantly higher in periodontal MSCs than in dental pulp. In contrast, pulp MSCs expressed higher levels of *CXCL14* and *RARRES1* than periodontal MSCs. They revealed that the MSCs in the dental pulp and periodontium, both as neural crest-derived cells, shared a common phenotype, and contained stem cells with high regenerative potential, showing overall homology, which was consistent with the previous study (Luan et al., 2009). The specificity of the respective niche is a potential source of the divergence in MSC function, which direct periodontal and dental pulp MSC to fibroblastic-like and osteogenic fate, respectively.

#### 3.2.2 Long Bone-Derived MSCs

The growth plate provides a continuous source of MSCs for endochondral ossification to construct a stromal compartment to maintain the expansion of bones and bone marrow space. *PTHrP*-expressing chondrocytes within the resting zone of the growth plate are considered one of the sources of SSCs, as they not only express a panel of skeletal stem/progenitor markers and possess characteristics of SSCs *in vitro* but also continuously form columnar chondrocytes that can generate osteoblasts and marrow stromal cells beneath the growth plate (Mizuhashi et al., 2018). In addition, the marginal chondrocytes around the growth plate behave as transient mesenchymal precursor cells, committed osteoblasts, and marrow stromal cells *in vivo*. Mizuhashi et al. (2019) performed single-cell sequencing on *Col2a1*-creER-marked chondrocytes in neonatal growth plates. They revealed that column-forming chondrocyte clusters from the growth plate of upper and lower region were marked by *Ucma* and *Prg4*, respectively; cluster abundant in tdTomato-WPRE was assigned as borderline chondrocytes between the upper and lower zone expressing *Pthrp* and *Cxcl14* and no hypertrophic markers. *PTHrP* ^+^ cells tracked non-self-renewing borderline chondrocyte subsets, which can give rise to short-lived osteoblasts and CXCL12-abundant reticular cells in the marrow cavity of long bone metaphysis (Mizuhashi et al., 2019).

MSCs in the periosteum are one of the major reservoirs of osteoprogenitor cells. Clinical and experimental data prove that the periosteum plays an essential role in postnatal bone growth, maintenance, and injury repair (Chaudhary et al., 2016; Debnath et al., 2018; Duchamp de Lageneste et al., 2018; Xiao et al., 2020). In adult mice, *Mx1*- and *aSMA*-labeled periosteal SSCs (P-SSCs) represent a unique lifelong sustainably regenerative stem cell group which serves as the main force for cortical bone regeneration and damage healing (Ortinau et al., 2019). There is evidence that P-SSCs contribute more to damage repair than bone marrow mesenchymal cells (BMSCs) (Worthley et al., 2015; Ortinau et al., 2019). With distinctive CCL5-dependent migration mechanism, *Mx1* ^*+*^ P-SSCs were rapidly recruited to injury area and generated osteoblasts, the number of which far exceeds of *Grem1* ^*+*^ bone marrow SSCs-derived (Ortinau et al., 2019). Besides, clusters of progenitor/stem cell and osteoblast were detected from scRNA-seq data of CTSK-mGFP+ periosteal mesenchymal cells. And in *Ctsk*-cre; mTmG reporter mice, Ctsk-cre-labeled *CD200* ^+^ *CD105* ^−^ cells were regarded as periosteal MSCs (PSCs) because of their capacity for “trilineage” differentiation, self-renewal and generation of the entire spectrum of CTSK-mGFP^+^ cells (bone, cartilage, stromal precursor/progenitor cells) (Debnath et al., 2018). PSCs specialize in intramembranous bone formation (Colnot, 2009), whereas P-SSCs display endochondral ossification and intramembranous bone formation.

BMSCs are the most commonly used stem cell in cell therapy clinical trials because of their ethical acceptability and accessibility. Researchers have characterized the heterogeneity and subpopulations of MSCs in different parts of the bone marrow cavity based on genetic fate tracking and single-cell sequencing (Severe et al., 2019; Tikhonova et al., 2019; Al-Sabah et al., 2020; Lu and Qiao, 2021). Baryawno et al. (2019) defined MSC clusters highly express *LepR* and *Cxcl12*, and two MSC-descendent osteolineage cells (OLC) subgroups express osteocalcin (*Bglap*). These two OLC subgroups are derived from distinct origins. The subpopulation composition in OLC-1 exhibited an osteolineage continuum from committed osteolineage LepR-MSC-4 in bone marrow, and expressed the key hematopoiesis-regulated cytokines, whereas OLC-2 mostly derived from bone, with no hematopoietic support potential. Besides, researchers have revealed temporal and spatial distinctions between BMSCs from the metaphysis (mpMSCs) and diaphysis (dpMSCs). Postnatal mpMSCs possessed multipotent properties. Clusters representing mpMSC, dpMSC, osteoprogenitor cells, osteoblasts, and proliferating BMSCs were identified in bone marrow. mpMSCs were placed in the center of pseudo-time trajectory, which directed to proliferating BMSC, dpMSC, and osteoprogenitor cell respectively (Sivaraj et al., 2021). *PDGFRα* ^*+*^ *β* ^+^ mpMSCs contained progenitors that gave rise to bone-forming osteoblast lineage cells, *LepR* ^+^ marrow stromal cells, and dpMSCs, whereas *PDGFRα* ^*+*^ dpMSCs from juvenile mice showed limited growth *in vitro* (Sivaraj et al., 2021). In addition, Chondrocytes and *Lepr* ^+^ BMSCs mediate longitudinal growth and transverse thickening of the bone before and after adolescence, respectively. (Shu et al., 2021) reported that osteoblasts are mainly emerged from Acan^+^ chondrocytes in the growth plate to realize bone lengthening before adolescence, whereas after adolescence, they primarily arise from *LepR* ^+^ BMSCs to achieve bone thickening.

#### 3.2.3 Craniofacial Skeleton-Derived MSCs

In contrast to long bone endochondral ossification, the craniofacial skeleton is mainly formed through intramembranous ossification. As the main growth centers for craniofacial bone development, calvarial sutures preserve the population of MSCs that support craniofacial bone repair. Gli1^+^ cells and *Axin2* ^+^ cells within the suture mesenchyme are the major MSC populations in the craniofacial bone and are regarded as suture stem cells (SuSCs) (Zhao et al., 2015; Maruyama et al., 2016). SuSCs are endowed with stem cell characteristics during calvarial development and homeostatic maintenance and are directly involved in injury repair and regeneration. Ablation of *Gli1* ^+^ and *Axin2* ^+^ cells leads to premature suture fusion (Zhao et al., 2015; Maruyama et al., 2016). However, the localization and responsible areas of *Axin2* ^+^ and *Gli1* ^+^ cells are different. *Gli1* ^+^ SuSCs are distributed throughout the mesenchyme suture and other osteogenesis regions within the suture, whereas *Axin2*-expressing cells are only limited to the middle part of suture mesenchyme, almost not adjacent to bone tissues. In addition, calvarial sutures contain SuSCs with the same immunophenotype as long bone-derived PSCs and share the same intramembranous osteogenesis pathway as PSCs (Debnath et al., 2018). Recently, researchers have successfully used *Gli1* ^+^MSCs to regenerate a functional cranial suture and ameliorate craniosynostosis in a mouse model (Yu et al., 2021). Hence, SuSCs are expected to bring new vitality to human craniosynostosis therapy.

## 4 scRNA-Seq on Cre-Targeted MSCS in Transgenic Mice

Lineage tracing is important in stem cell research, which through the Cre recombinase (Cre)-*loxP* system permanently marks specific cells and tracks the proliferation, differentiation, and migration activities of specific cells and their descendants *in vivo*. Several endogenous osteogenesis stem/progenitor cell populations were marked by the expression of *Gli1, Osx, Ctsk, Pdgfrβ*, etc. In recent years, researchers have used lineage tracing combine with fluorescence-activated cell sorting techniques to tag and sort MSCs based on a series of markers, and subsequently performed scRNA-seq on them, thereby realizing the reconstruction of cell development trajectories and parsing the regulation of fate-determining gene expression (Table 2).

**TABLE 2:** Lineage-tracing mouse transgenic lines with stem/progenitor markers for scRNA-seq.

Driver	Representative cre-marked cells	Single-cell sequencing object	Subclusters	Time points of induction	Reference
Gli1-CreERT2; tdTomato	Metaphyseal mesenchymal progenitor cells	TdTomato^+^CD45^–^Ter119^–^CD31^–^ cells	Osteoblasts, preosteoblasts, chondrocyte-like osteoprogenitors and marrow adipogenic lineage progenitors	Three consecutive days at 4 weeks old	Shi et al. (2021)
Pdgfrβ-CreERT2; Rosa26-mTmG	Pdgfrβ+BMSCs	GFP^+^cells	BMSCs, chondrocytes, smooth muscle cells, fibroblasts, hematopoietic cells	Postnatal day 1–3	Bohm et al. (2019)
Ctsk-cre; mTmG	Periosteal stem cells	Metaphyseal CTSK-mGFP^+^ cells	Progenitor/stem cells, osteoblasts, *Ly6a* and *Acta2* expressing cells	N/A	Debnath et al. (2018)
Cxcl12GFP/+; Cxcl12-creER; R26RtdTomato	Cxcl12-creER+stromal cells	Cxcl12-GFP^+^ cells	Stromal (reticular cells and pre-osteoblasts), endothelial, periosteal and cells in cell cycle, clusters enriched for mitochondrial and ribosomal genes	Postnatal day 21	Matsushita et al. (2020)
Lepr-cre; LoxP-tdTomato	LepR+ cells	Lepr-tdT cells	Osteo-primed *LepR* ^+^ cells and adipocytic-primed *LepR* ^+^ cells	N/A	Tikhonova et al. (2019)

*Gli1*, which encodes the transcriptional key effector of Hedgehog (Hh), serves as a primary marker of MSCs. *Gli1* ^+^ cells continuously replenish osteoblasts for bone development and repair. Within craniofacial sutures, *Gli1* ^+^ cells were proposed as the major MSC population for craniofacial bone, responsible for the growth and injury repair. In postnatal mice, *Gli1* ^+^ cells inhabiting underneath the growth plate, were called metaphyseal mesenchymal progenitor cells, which can generate osteoblasts, adipocytes, and stromal cells *in vivo* and express *Pdgfrα*, *LepR*, and other MSC marker genes (Shi et al., 2017). *Gli1* ^+^ cells in the periodontal ligament of adult mouse molars can form the periodontal ligament, cementum, and alveolar bone. They support the renewal and injury repair of periodontal tissue, and their activity is modulated by canonical Wnt signaling (Men et al., 2020). In *Gli1*-CreERT2; tdTomato mice, metaphyseal mesenchymal progenitor cells (*Gli1* ^+^ cells) dissociated from metaphyseal trabecular bone, are clustered into four subsets: osteoblasts, pre-osteoblasts, chondrocyte-like osteoprogenitor, and marrow adipogenic lineage progenitors, among which chondrocyte-like osteoprogenitor is a potential target for widespread bone anabolic drug, teriparatide, and expresses the high levels of growth-related factors such as Hh target genes and *IGF-1* (Laron, 2001; Shi et al., 2021).

PDGFRβ is involved in the maintenance of immature and proliferative states for skeletal stem/progenitor cells (SSPCs). *Pdgfrβ*-traced cells were displayed in the growth plate, metaphyseal, periosteal, BMSCs, and perivascular space inhabited by osteo-primed multipotential stem cells (Bohm et al., 2019). In *Pdgfrb*-CreERT2; Rosa26-mTmG mice, long bone-derived GFP^+^ cells belonged to three major sub-populations (BMSCs, chondrocytes, and smooth muscle cells) and two minor subsets (fibroblasts and hematopoietic cells). Trajectory analysis and *in vitro* culture of GFP^+^ BMSC support *Pdgfrβ* ^+^ BMSCs possessing trilineage differentiation potential (Bohm et al., 2019). PDGFRβ promotes osteogenesis and angiogenesis in the postnatal period and activates SSPC in injury. Following a fracture, reparative SSPCs in the periosteal, endosteal and perivascular spaces were activated and recruited under the regulation of Pdgfrβ signaling. In *Pdgfrβ* ^+^ cell-ablated mice, the length of the femur and the number of Osx^+^ osteoprogenitors decreased (Liu et al., 2013). In contrast, overexpression of human *Pdgfrβ* led to increased vessels, *Pdgfrβ* ^+^ mpMSCs, and osteoprogenitors (Bohm et al., 2019).

Osteoclasts secrete a cysteine protease called cathepsin K (CTSK) which plays an important role in the degradation of matrix collagen during bone resorption. Several studies have shown that *Ctsk* ^+^ cells possess progenitor/stem cell properties (Han et al., 2019). Yang et al. (2013) illustrated that chondroid neoplasms originate from the *Ctsk*-Cre labeled cell population in perichondrial groove of Ranvier, which exhibits markers and functional characteristics comparable to mesenchymal progenitors. Further studies have shown that *Ctsk* ^+^ periosteal cells contain stem cell populations that mediate intramembranous osteogenesis. ScRNA-seq performed on *Ctsk* ^+^ cells derived from Ctsk-cre; mTmG reporter mouse femurs generated four clusters: expressing osteoblast markers, MSCs marker, stem cell antigen 1 (*Sca1*), and *Acta2*, respectively. Monocle-inferring differentiation trajectory showed that MSCs markers were expressed in the prophase of trajectory map containing periosteal stem cells, which supports the existence of stem/progenitor cells among *Ctsk* ^+^ cells (Debnath et al., 2018).

As a chemotactic protein, CXCL12 serves as an influential regulator of the musculoskeletal niche. It predominantly acts through the G-coupled protein receptor (CXCR4) and is involved in the conscription, location, growth, and fate determination of hematopoietic and MSCs in the musculoskeletal system (Wright et al., 2005; Hosogane et al., 2010; Fujio et al., 2011). Compared with immature MSCs, alkaline phosphatase-expressing differentiated osteogenic cells displayed decreased *Cxcl12* expression levels (Gilbert et al., 2019). CXCL12 is involved in the early differentiation of pre-osteoblast MSCs, but as the cell matures, the scale of involvement of CXCL12 gradually reduced. As a result, if CXCL12 signaling is disrupted, reduced migration and differentiation may ensue, potentially leading to a reduction in the quantity and viability of progenitor cells (Zhang et al., 2008; Guang et al., 2013). *CXCL12*-GFP^+^ BM cells from *Cxcl12* ^GFP/+^; *CXCL12*-creER; R26RtdTomato mice were divided into three categories, stromal, endothelial, and periosteal at P28. *CXCL12* ^+^ stromal cell consisted of three clusters, two of which were reticular cells and osteogenic progenitors, expressing pre-adipocyte markers (*Adipoq* and *LepR*) and pre-osteoblast markers (*Alpl* and *Postn*), respectively. Upon the induction of injury, quiescent CXCL12-creER^+^ BMSCs were recruited to the cortical defect, converted into SSC-like state through the regulation of canonical Wnt signaling components, and then differentiated into cortical bone, which does not occur in homeostasis (Matsushita et al., 2020).

As an endocrine hormone, leptin participates in energy metabolism and regulates the promotion and inhibition of MSCs osteogenesis. Previous studies have indicated that leptin enhances BMSC adipogenesis at the expense of osteogenesis, whereas it can promote osteogenesis of cultured MSC in other study (Thomas et al., 1999; Ambati et al., 2010). LepR is a marker that highly enriched BMSCs (Zhou et al., 2014). With trilineage differentiation potential, *LepR*-expressing cells can give rise to osteocytes, chondrocytes, and adipocytes through *in vitro* culture and xenotransplantation. Lineage tracing showed that *LepR* ^+^ cells are not only the main source of osteocytes and adipocytes in the adult bone marrow but also a cell reservoir of endochondral osteogenesis during embryonic and postnatal bone formation (Yue et al., 2016). Shen et al. (2021) revealed that an osteogenic growth factor, osteolectin, marked peri-arteriolar rapidly dividing *LepR* ^+^ osteogenic progenitor cells, which increased after injury and depleted during aging. During injury repair, *LepR* ^+^ cells form new chondrocytes and osteoblasts, whereas leptin inhibits bone regeneration by negatively regulating bone marrow osteogenesis (Zhou et al., 2014). *LepR* ^+^ MSCs labeled in LepR-cre; LoxP-tdTomato mice possessed potential for both adipogenesis and osteogenesis. The subpopulation clustered by *LepR* ^+^ cells scRNA-seq analysis showed high levels of adipogenesis- and osteogenesis-associated markers, respectively (Tikhonova et al., 2019). Furthermore, osteo-primed *LepR* ^+^ cells were highly correlated with multipotential human *CD45* ^−^ *CD271* ^+^ BMSCs (Ghazanfari et al., 2016), and adipocytic-primed *LepR* ^+^ cells were the main pro-hematopoietic factor supplier in the BM niche. In response to stress hematopoiesis, adipogenesis-related pathways in *LepR* ^+^ cells significantly increased, and the expansion of adipocytes was observed after bone marrow insult. In contrast, the expression of ossification-related genes was decreased (Tikhonova et al., 2019).

Genetic lineage tracing based on Cre-loxP system is powerful and widely used in tracking stem cell differentiation and fate. However, single MSC markers are not specific to stem cell populations, making the reliability of this system still debatable. Dual-recombinase-activated lineage tracing (DeaLT) technology introduces the Dre-rox recombination system into the traditional Cre-loxP recombination system, allowing the more precise definition and tracking of cell populations, significantly improving the resolution of lineage fate maps. Different permutations and combinations of Dre-rox and Cre-loxP in the genome can produce different effects, mainly including the following three type: 1) Cre^+^Dre^+^, requires two promoters to drive Dre and Cre to recombination, mainly labeling the double-positive cells. 2) Cre^+^Dre^−^ or Cre^−^Dre^+^, highlight the exclusivity of the two recombination systems, mainly labeling single marker positive cells. 3) Cre^+^Dre^+^/Cre^−^Dre^+^ or Cre^+^Dre^+^/Cre^+^Dre^−^, mark the double-positive cells, and the cross-section is read out by one of the two markers. More detailed review information has been presented by Zhao and Zhou et al. (2019). This technology has been applied to reveal the developmental origin of hepatic vasculature and the cell fate of club cells, AT2 cells and bronchoalveolar stem cells in the process of lung repair and regeneration (Zhang et al., 2016; Liu et al., 2020). However, few MSC-related studies are using DeaLT technology (Shu et al., 2021). The combined application of DeaL technology and single-cell technology will bring more precise and reliable interpretations on the heterogeneity of MSCs, and facilitate understanding of the regulatory network that controls cell state, which exhibits extensive application prospects and warrant further exploration.

## 5 Single-Cell Analysis of MSC in Response to Microenvironment

The MSC niche supports the self-renewal and multi-lineage differentiation of MSCs. Distinct cell populations in the stem cell microenvironment provide signaling molecules for properly maintaining the proliferation and differentiation of stem cells, which are necessary for tissue stability. MSCs are heterogeneous, and the surrounding niche cells have different metabolic states. MSC will adapt to different microenvironments and perform specific functions through reprogramming. At present, with the help of single-cell sequencing technology, scientists can obtain gene expression profiles of MSC and surrounding niche cells, dissect the metabolic information of different cell types, evaluate the interaction between MSC and the microenvironment, and then study the genetic and environmental factors of MSC at the single-cell level.

### 5.1 Tooth Niche Cell Regulates MSCs Homeostasis

**FIGURE 3:**
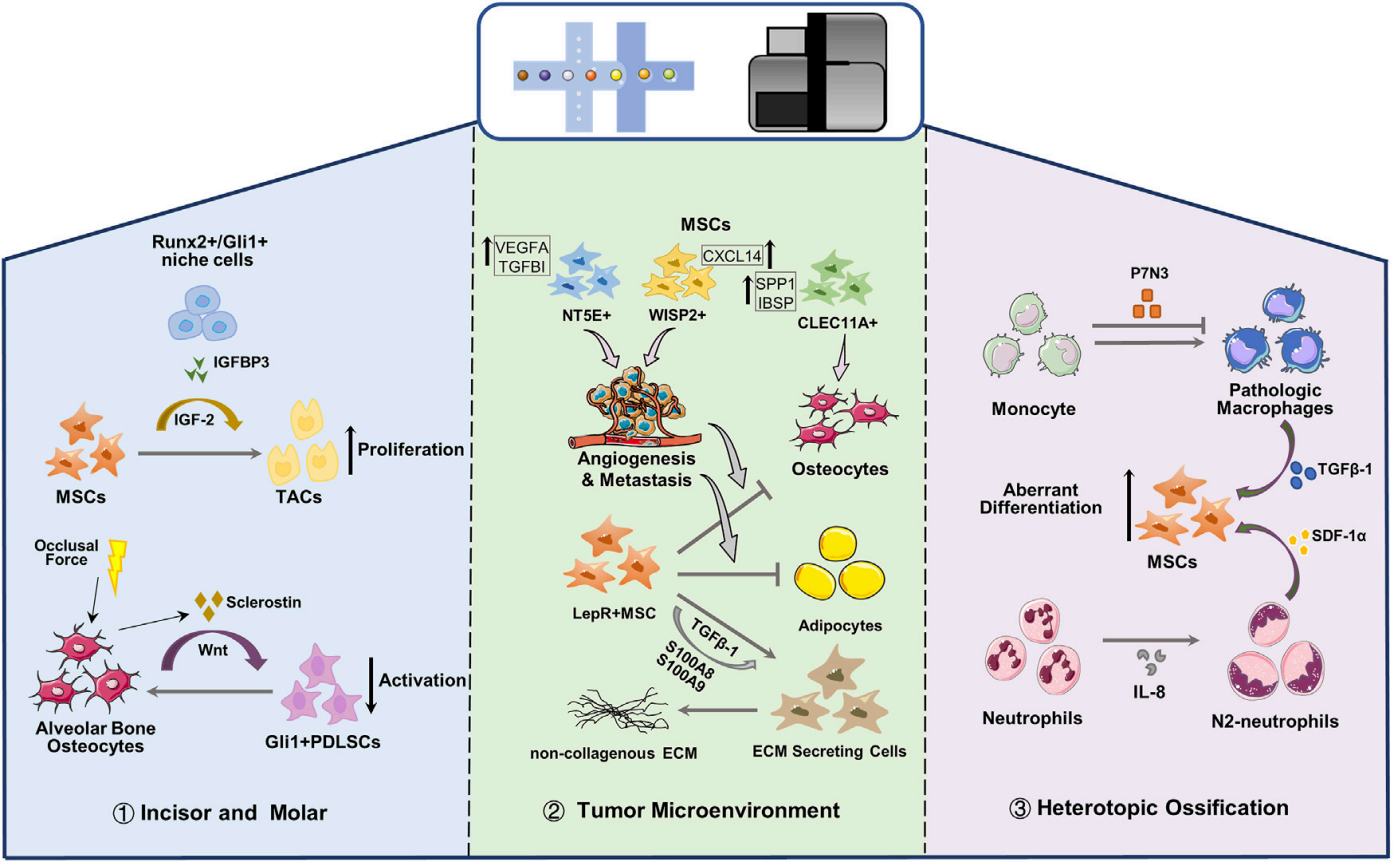
Single cell reveals the crosstalk between MSCs and microenvironment. MSC, Mesenchymal stem cells; TAC, transit-amplifying cells; PDLSC, periodontal ligament stem cells; ECM, extracellular matrix.

In the dental mesenchyme, *Gli1* is typical marker of MSCs in the mouse incisor and RUNX2 is an important transcription factor that regulates bone and tooth development. In adult mouse incisor, most *Gli1* and *Runx2* co-expressing cells were clustered into a subgroup located proximal region of dental mesenchyme and were adjacent to transit-amplifying cells, which suggested niche cell identity (Chen et al., 2020). Runx2^+^/Gli1^+^ niche cells secrete Insulin-like growth factor-binding protein 3, thereby activating the exp-mediated IGF2 signaling pathway and coordinating the transition of MSCs to transit-amplifying cells, thereby regulating the proliferation and differentiation of transit-amplifying cells, maintaining the homeostasis of mesenchymal tissue, and controlling the growth rate of incisors (Chen et al., 2020). In addition, studies have shown that osteogenesis in long bones or alveolar bone is governed by sclerostin and mechanical loading (Robling et al., 2008; Men et al., 2020). For the periodontal ligament, the activation and maintenance of *Gli1* ^+^ PDLSCs are modulated by canonical Wnt signaling, whereas alveolar bone cells that secrete sclerostin inhibit Wnt signaling and negatively modulate PDLSC activity (Men et al., 2020). Physiological occlusal force controlling the expression of sclerostin is indirectly involved in the activity of PDLSCs, and this mechano-response is essential for *Gli1* ^+^ PDLSCs activation (Figure 3).

### 5.2 The Lineage Transition of MSCs in Senescent

Aging is accompanied by the accumulation of genetic damage, leading to changes such as mutation, telomere shortening, cellular senescence, and stem cell depletion, etc. As the source of cells in bone marrow, MSC maintains bone metabolism. However, with the increase of age, MSCs tend to differentiate into adipocytes and decreased osteogenesis, leading to an increased risk of aging-related bone diseases for the elderly, and taking a long time to heal fractures (Woods and Guezguez, 2021). Experiments have shown that senescent MSCs exhibit reduced clonal formation and proliferation capabilities compared to young donor-derived MSCs *in vivo* and lineage tracing *in vitro* demonstrated an age-dependent lineage transition between osteogenesis and adipogenic differentiation (Sui et al., 2020). To define MSCs at young, adult, and aging stages, Zhong et al. (2020) performed single-cell sequencing of *Col2* ^+^ cells in the long bones of 1, 3, and 16-month-old mice, and divided the MSCs into three subgroups. Pseudotime trajectory inferred that the early mesenchymal progenitors (EMPs) in the three subgroups are the ancestors of two other subgroups expressing *Sca1*, *Thy1* and *Cd34*, and the next ones are the intermediate mesenchymal progenitor cells and late mesenchymal progenitors respectively. Consistent with the results of CFU-F, the number of MSCs with osteogenic/adipogenic capabilities in the bone marrow of aging mice shrank sharply, while the number of adipocytes greatly increased. In young and adult mice, the intermediate and late mesenchymal progenitor clusters are concentrated at the starting point of differentiation. In the elderly, the loss of intermediate mesenchymal progenitor was observed. MSCs were prone to differentiation, and adipocyte markers such as *CBPA* and *LPL* were also highly expressed, which indicated that aged EMPs not only decrease in number but also drift towards a more adipogenic state. The expression of adult MSC marker *Lepr* in 16-month-old EMPs was significantly higher than that of 1 month, which was consistent with the previously observations that *Lepr*-Cre labeled MSCs only appeared in the bone marrow of adult mice, but not in young mice (Zhou et al., 2014; Zhong et al., 2020).

### 5.3 MSCs Under the Crosstalk With Tumor Microenvironment

Due to the aggressive growth of tumors, MSCs with regenerative capacity are recruited into lesions for tissue repair. In addition, the increased acidification, nutritional deficiency, inflammatory, hypoxia, and tumors microenvironment (TME) lead MSCs to accumulate in lesions. CXCR4 and Matrix Metalloproteinases 2 (MMP2) participate in the migration process of MSC to TME (Song and Li, 2011). After being recruited to the tumor, the crosstalk between MSCs and cancer cells reshapes the expression pattern of MSCs, thereby changing the direction of differentiation and adapting to the TME by acquiring certain functions, and this crosstalk also changes the metastatic potential and invasion efficiency of cancer cells (Whiteside, 2018). BMSCs promote breast carcinoma cells to produce lysyl oxidase, leading to increased metastatic potential and invasive activity of cancer cells (El-Haibi et al., 2012). In TME, MSCs are induced to differentiate into fibroblasts or myofibroblasts, which stabilize tumor tissue and enhance chemotherapy resistance and cancer stemness (Quante et al., 2011). TME-derived TGF-β1 activates MSCs to form cancer-associated fibroblasts-like phenotype. BMSC secrete a series of tumor suppressor factors such as DKK-1/3, interferon, CXCL10, IL12, etc. Dickkopf-1 (*DKK-1*) as a negative regulator of the Wnt/β-catenin pathway endow MSCs with anti-proliferation and anti-tumor effects on erythroleukemic and breast cancer cells (Thiago et al., 2010; Kim et al., 2015). BMSC derived-extracellular vesicles inhibit proliferation and promote apoptosis of liver carcinoma, Kaposi’s sarcoma and ovarian tumor cell line (Takahara et al., 2016). The distinction between cancer-promoting MSCs and anti-tumor MSCs may be related to its heterogeneity in TME and the type of cancer. Therefore, it is necessary to explore the heterogeneity of MSC in TME.

Zhou et al. (2020) performed scRNA-seq on osteosarcoma lesions, and the identified clusters are all the progeny of MSC or hematopoietic stem cells. Malignant osteoblastic cells in osteosarcoma can be derived from any cell type in osteogenic lineage of MSCs. MSC cells characterized by *MMe*, *THY1* and *CXCL12* are divided into three subgroups (*NT5E* ^+^, *WISP2* ^+^ and *CLEC11A* ^+^), and are present in different types of osteosarcoma lesions (Figure 3). *NT5E* ^+^ MSCs is mainly observed in chondroblastic osteosarcoma lesions which stimulate the angiogenesis and metastasis of osteosarcoma cells; MSCs with high expression of *WISP* and *CXCL14* are regarded to promote the metastasis of osteosarcoma cells and the proliferation of MSCs; *CLEC11A* ^+^ MSCs mainly exist in osteoblast osteosarcoma lesions with highly expressing osteoblast differentiation markers (*SPP1* and *IBSP*). Although more efforts are required to reveal the influence of MSCs on malignant osteoblasts and chondrocytes in osteosarcoma lesions, gene expression data suggest that MSCs can help osteosarcoma cells to metastasize or proliferate (Zhou et al., 2020).

MSCs are the main members of the bone marrow niche, which can regulate hematopoietic stem cells and supply hormones and nutrition, and are closely related to various myeloid malignancies. The bone marrow niche under myeloid disease will reshape the transcription of MSCs, enabling lineage transfer and function reprogramming, and making it a potential target for the treatment of myeloid diseases. MSC has become a promising therapeutic tool for many clinical applications due to its unique immunomodulatory properties, which can secrete cytokines, reduce inflammation and cell apoptosis, and promote the proliferation of stem progenitor cells in endogenous tissues and organs (Sivanathan and Coates, 2018; Qin and Zhao, 2020; He et al., 2021b). In primary leukemia, the osteogenic Lepr-MSCs decreased, and the pre-osteoblasts in the OLC subgroup increased significantly (Baryawno et al., 2019). The osteogenic genes in Lepr-MSCs and OLC were down-regulated, and the genes that inhibit bone formation and calcification were up-regulated. The adipogenic genes in *Lepr*-MSCs were significantly down-regulated, resulting in the obstructed development of osteogenic lineage cells (Figure 3), the damaged adipocytes niche, and the loss of hematopoietic stem cells niche factors, leading to a niche that is not conducive to the production of normal blood cells (Baryawno et al., 2019). Besides, Adipogenic and osteogenic *Lepr* ^+^ MSCs are considered to be the main driving factors of myelofibrosis in myeloproliferative neoplasms (Koschmieder and Chatain, 2020). In Thrombopoietin-induced BM fibrosis, *LepR* and *Gli1* labeled adipogenic and osteogenic MSC clusters significantly up-regulate non-collagenous extracellular matrix (ECM) specific genes, and down-regulate MSC markers, which leaded to the loss of progenitor cell status and were reprogrammed into ECM secreting cells (Figure 3) (Leimkuhler et al., 2021). Expression profiles and pseudo-time trajectories showed that adipo- and osteo-primed MSC clusters exhibited high level adipogenic (*Adipoq*) and osteogenic (*Sp7*, *Ibsp*) signature, and were inferred to be pluripotent precursor cells. With the development of fibrosis, MSC marker genes and hematopoietic support genes were significantly down-regulated in all MSC clusters, while secreted factors (*S100A8*, *S100A9*) and genes related to ECM synthesis were significantly up-regulated, indicating that they lost the ability to support hematopoiesis and excessive maldifferentiation, resulting in exorbitant deposition of ECM in the bone marrow (Leimkuhler et al., 2021). MSC-mediated inflammation is the main driving force for the transition of the fibrotic lineage, especially S100A8/S100A9 pro-inflammatory factors expressed by MSCs and the TGF-b signaling pathway. The novel small-molecule tumor suppressor, Tasquinimod, can inhibit S100A8/S100A9 signal transduction and effectively ameliorate fibrosis and the phenotype of myeloproliferative neoplasms (Leimkuhler et al., 2021).

### 5.4 Injury-Induced Niche Regulates MSC Behavior

In the musculoskeletal trauma site, inflammation niches recruit and orchestrate MSCs and immunocytes. MSCs attempt to regenerate tissues according to the prompts of inflammation and the immune microenvironment and sometimes lead to the abnormal cell fate of MSCs, resulting in heterotopic ossification (HO) (Figure 3). Fiber/adipogenic progenitor, a population of pluripotent stem cells, are the main contributors of HO, expressing PDGFRα and other MSC maker, which exhibits potential for adipogenic, chondrogenic and osteogenic differentiation (Lees-Shepard et al., 2018; Eisner et al., 2020). Fibrous/adipogenic progenitor cells can produce pivotal growth factors and matrix or matrisome proteins, such as IGF-1, TGF-β, collagens, integrins, etc., which impact the cellular physiology during injury, disease and homeostasis (Biferali et al., 2019; Theret et al., 2021). Recently, Sorkin et al. (2020) used single-cell transcriptome and trajectory analyses to identify different monocyte/macrophage subpopulations after injury and their dynamic changes in the different stages of inflammation after injury. They discovered a cluster of Pdgfrα labeled-stromal cells, and the abnormal differentiation of chondrogenic progenitor cell was predetermined 3 days after injury. After burn/tenotomy injury, p7N3 (transforming growth factor-1 expression regulator) treatment altered macrophage subset phenotypes. In addition, the expression levels of chondrogenic and osteogenic markers (*Sox9*, *Runx2*, *Acan* and *Col2a1*) of the stromal cell clusters in the p7N3 treatment groups are lower than control. They revealed that monocytes/macrophages expressing transforming growth factor-1 play an important role in endochondral osteogenesis-driven mesenchymal chondrogenic progenitor cell abnormal differentiation during HO progression. (Sorkin et al., 2020). Furthermore, Cai et al. (2021) found that a certain level of interleukin-8 (IL-8) polarizes neutrophils toward the N2 phenotype, which initiates bone regeneration by secreting stromal cell-derived factor-1α and initiating its downstream cascade reaction to mediate BMSC recruitment and differentiation, thereby inducing ectopic endochondral ossification.

## 6 Conclusion and Future Perspectives

In this review, we summarized the heterogeneity of human and mouse MSCs at single-cell resolution from embryo development to adulthood and emphasized subsets with osteogenic potential. During the initial stage of embryonic limb buds, the subpopulations of limb bud MSCs exhibit continuous marker expression patterns and uniquely respond to morphogenesis gradient signals and bone-forming signals, thereby displaying distinct differentiation states and osteogenic activity in different limb bud regions. Among them, subgroups marked by transient high expression marker genes require much weighted attention, in which phased transcription patterns in these subgroups have important consequences for musculoskeletal development. More nuanced analyses are needed to fully understand the changes in cell transcription during the development process, as well as the impact and significance of its existence. ScRNA-seq has identified stem/progenitor cell subsets with osteogenic potential in various cell types of hard tissues, including dental pulp/periodontal stem cells, bone marrow/periosteal stromal cells, chondrocytes, and craniofacial suture mesenchymal stem cells (Zhao et al., 2015; Mizuhashi et al., 2018; Tikhonova et al., 2019; Pagella et al., 2021). Unlike MSCs, SSCs identified in bone marrow have no adipogenic ability, and exclusively generate osteogenic lineages. Although many of the above-mentioned osteogenic cell subsets are called as MSCs, they may still retain adipogenic and myogenic potential. At present, the term, “skeletal stem cells”, is under continuous refinement, the relationship and underlying molecular differences between SSCs and MSCs cannot be clearly defined yet. Similar to hematopoiesis, osteogenic stem cells also depend on the mediation of niche-derived signals, extracellular matrix, and cytokines. However, compared with the hematopoietic stem cell niche, studies on MSC microenvironment are relatively lacking. In addition to inflammatory microenvironment, the interaction between stem cells and surrounding niche cells is also largely unelucidated, when the crucial phase transitions of bone-forming pattern, especially during bone growth spurt in puberty and aging-related osteoporosis.

Besides scRNA-seq, single-cell technology includes single-cell ChIP-seq and ATAC-seq, etc., which allow the detection of intercellular heterogeneity and can comprehensively reveal interactions between cell movement, signal transduction pathways, transcription factors, and genome chromatin packaging, to deeply characterize stem cell populations in various tissues. Nonetheless, the main obstacle of single-cell technology is the loss of all the spatial information of the original cells. Recently emerging spatial transcriptome technology has swept the obstacle away, which allows the analysis of transcriptome information while preserving the spatial location of the tissue section (Liao et al., 2021). The integration of spatiotemporal information and single-cell transcriptomics pave the way for identifying the pivotal distinctions between cell subtypes and in-depth analysis of developmental tissues and is expected to bring new insights into MSCs.

## Abbreviations

LMPs, Limb Bud Mesenchymal Progenitors; POC, Primary Ossification Center; SSCs, Skeletal Stem Cells; OCPs, Osteo-chondrogenic Progenitors; MSCs, Mesenchymal Stem Cells; mSSCs, Mouse Skeletal Stem Cell; WPC, Weeks Post Conception; eSSPCs, Embryonic Skeletal Stem/Progenitor Cells; NCDCs, Neural Crest-derived Cells; PDLSCs, Periodontal Ligament Stem Cells; P-SSCs, Periosteal Skeletal Stem Cells; BMSCs, Bone Marrow Mesenchymal Cells; PSCs, periosteal Stem Cell; OLCs, Osteolineage Cells; mpMSCs, Mesenchymal Stem Cells From The Metaphysis; dpMSCs, Mesenchymal Stem Cells From The Diaphysis; SuSCs, Suture Stem Cells; MMPs, Metaphyseal Mesenchymal Progenitor Cells; BMP, Bone Morphogenetic Protein; CTSK, Cathepsin K; Sca1, Stem Cell Antigen 1; CXCL12, C-X-C Motif Chemokine Ligand 12; EMPs, Early Mesenchymal Progenitors; TME, Tumors Microenvironment; ECM, Extracellular Matrix; SSPCs, Skeletal Stem/progenitor Cells; HO, Heterotopic Ossification.
